# RETRACTION: Role of the MUC1‐C Oncoprotein in the Acquisition of Cisplatin Resistance by Urothelial Carcinoma

**DOI:** 10.1111/cas.70016

**Published:** 2025-02-11

## Abstract

**RETRACTION:** K. Shigeta, M. Hasegawa, E. Kikuchi, Y. Yasumizu, T. Kosaka, R. Mizuno, S. Mikami, A. Miyajima, D. Kufe and M. Oya, “Role of the MUC1‐C Oncoprotein in the Acquisition of Cisplatin Resistance by Urothelial Carcinoma,” *Cancer Science* 111, no. 10 (2020): 3639–3652, https://doi.org/10.1111/cas.14574.

The above article, published online on 16 July 2020 in Wiley Online Library (wileyonlinelibrary.com), has been retracted by agreement between the journal Editor‐in‐Chief, Masanori Hatakeyama; the Japanese Cancer Association; and John Wiley & Sons Australia Ltd. The retraction has been agreed upon due to several instances of duplications of western blot bands observed between Figures 2E and 3B; 4A and 4D; S1b and S3a; and within S1d. The authors commented that this was due to inadvertent errors and provided some data including replacement images. However, the data supplied did not meet acceptable standards for raw data and the editors were not satisfied with the explanation provided. Due to the extent of the errors, the editors have lost confidence in the results and conclusions of this study. The authors disagree with the retraction.

**RETRACTION:** K. Shigeta, M. Hasegawa, E. Kikuchi, Y. Yasumizu, T. Kosaka, R. Mizuno, S. Mikami, A. Miyajima, D. Kufe and M. Oya, “Role of the MUC1‐C Oncoprotein in the Acquisition of Cisplatin Resistance by Urothelial Carcinoma,” *Cancer Science* 111, no. 10 (2020): 3639–3652, https://doi.org/10.1111/cas.14574.

The above article, published online on 16 July 2020 in Wiley Online Library (wileyonlinelibrary.com), has been retracted by agreement between the journal Editor‐in‐Chief, Masanori Hatakeyama; the Japanese Cancer Association; and John Wiley & Sons Australia Ltd. The retraction has been agreed upon due to several instances of duplications of western blot bands observed between Figures 2E and 3B; 4A and 4D; S1b and S3a; and within S1d. The authors commented that this was due to inadvertent errors and provided some data including replacement images. However, the data supplied did not meet acceptable standards for raw data and the editors were not satisfied with the explanation provided. Due to the extent of the errors, the editors have lost confidence in the results and conclusions of this study. The authors disagree with the retraction.

